# The impact of knowledge and attitudes on adherence to tuberculosis treatment: a case-control study in a Moroccan region

**Published:** 2012-06-28

**Authors:** Nabil Tachfouti, Katia Slama, Mohammed Berraho, Chakib Nejjari

**Affiliations:** 1 Laboratory of Epidemiology, Clinical research and Community health. Faculty of Medicine – Fez Morocco; 2 French School of Public Health (EHESP) – Rennes – France

**Keywords:** Tuberculosis, Non-adherence, DOT, knowledge, attitudes, treatment, Morocco

## Abstract

**Background:**

Although tuberculosis (TB) care is provided free of charge in Morocco, a high number of patients voluntarily interrupt their treatment before the end. Treatment Default is a major obstacle in the fight against the disease. The purpose of this study was to describe the impact of knowledge and attitudes toward TB on treatment adherence.

**Methods:**

Case-control study of 290 TB patients (85 defaulters and 205 controls). A defaulter was defined as a TB patient who interrupted treatment for two months or longer. Socio-demographic measurements, knowledge and attitude were collected by face to face anonymous questionnaire. Khi-square test was conducted to examine differences in TB attitudes and knowledge according to treatment adherence.

**Results:**

The mean age of participants was 31.7 ± 12.0 years. Monthly income was under 2000 MAD (180 €) for 82% of them. Over sixty four percent were illiterate or had a basic educational level. Microbial cause was known by 17.2% respondents; 20.5% among adherent patients versus 9.4% (p=0.02). The fact that the disease is curable was more known by adherent patients: 99.0% versus 88.2% (p < 0.01). Eighty tree per cent of patients had been informed about treatment duration and consequences of not completing treatment: 89.0% among adherent patients versus 69.7% (p<0.001). The main reason evoked for defaulting was the sensation of being cured (72.9% of defaulters).

**Conclusion:**

This study shows a poor knowledge on TB especially among non adherent patients. This finding justifies the need to incorporate patient's education into current TB case management.

## Background

Tuberculosis remains a major public health problem in the world. It affects one third of the world's population [1]. According to World health Organization (WHO) estimations, approximately 9.4 million new cases were reported in 2009. WHO estimates that the largest number of new TB cases in 2009 occurred in the South-East Asia Region, which accounted for 35% of incident cases globally. However, the estimated incidence rate in sub-Saharan Africa is nearly twice that of the South-East Asia Region with over 350 cases per 100 000 population. An estimated 1.7 million people died from TB in 2009, the highest number of deaths was in the Africa Region. [2].

To stop the spread of the disease, treatment of active pulmonary TB patients remains the most effective strategy since a contagious person will infect up to 15 people every year if left untreated [3]. A World Health Assembly resolution invigorated the TB control efforts in 1991 and the internationally recommended control therapy, later named DOTS (Directly Observed Therapy Short-course), was launched in 1994 [4].

However, interruption in tuberculosis treatment still remains the major barrier to its control and is the most important challenge for control of TB. Inability to complete the prescribed regimen is an important cause of treatment failure, drug resistance and continuous transmission of infection [5]. Adherence to the long course of TB treatment is a complex phenomenon with a wide range of factors impacting on treatment taking behavior.

Lack of knowledge about the disease and stigmatization causes underutilization of the services, delay in seeking diagnosis, and poor treatment adherence [6]. Several international studies have reported poor knowledge, attitudes and practices about TB [7]. Non-adherence to treatment often results from inadequate knowledge or understanding of the disease and its treatment [8].

Studies in different parts of the world revealed misconceptions and limited knowledge about the disease and its treatment [9]. For example, a study in Egypt revealed that the significant risk factors for treatment failure were non-adherence to treatment, due to deficient health education and poor patient knowledge about the disease [10].

In Morocco, TB is highly prevalent; it affected more than 26.000 people in 2009, with an incidence of approximately 82 new cases per 100.000 populations (Health Ministry statistics). In 2008, detection rate was 80% and the estimated proportion of relapses and treatment failures was 15% [11].

The Moroccan government is committed to fight TB, strengthened by the adoption of the National Strategic Plan 2006-2015, which aims to enhance the therapeutic delivery system to TB patients and accelerate the reduction of the incidence of smear positive cases. Morocco is one of the few middle-income countries achieving Millennium Development Goals (MDGs) and the Stop TB Partnership [12].

National TB treatment guidelines in 2007 and 2008 recommended a Category I treatment regimen-2 months of isoniazid, rifampin, pyrazinamide, and streptomycin followed by 4 months of rifampin and isoniazid (2SHRZ/4RH)-for new smear-positive cases and a Category II regimen-2HRZES/1RHEZ/5RHE (E = ethambutol)-for retreatment cases [13]. TB care is provided free of charge by the Ministry of Health. Regional TB programs are divided into sectors, and each sector has a public health center (CDTMR) staffed by a specialist in TB care. TB clinical care is provided at CDTMR, while TB medications are delivered via DOTS at local clinics or dispensaries. DOTS consist on home-based observed treatment.

Despite the absence of fees to pay for treatment, a high number of new smear positive cases interrupt voluntarily their treatment before the end. Treatment default is a major obstacle in the fight against TB, and was estimated around 10% in 2009 [3]. However, the association between TB knowledge and treatment default has not yet received much attention. The purpose of this study was to describe the impact of TB knowledge and attitudes on treatment adherence among Fez community.

## Methods

### Setting

The study was conducted in ten tuberculosis control units (TCUs) in public primary and respiratory care centers in Fez city. The TCUs were selected based upon case load and nurses’ willingness to participate. There cover more than 50% and reflect socio-demographic characteristics of Fez TB patients.

### Design

Case-control study of 290 TB patients (85 defaulters and 205 controls). A defaulter (case) was defined as a TB patient who interrupted treatment for two months or longer before the end of the treatment period [13]. A non-defaulter (control) was defined as a TB patient diagnosed during the same time period, from the same TCUs, following the same treatment protocol, but who completed the whole treatment regimen. Cases and controls were matched by sex and age; two non-defaulters were selected for each case of default.

### Study population

Study included all TB patients aged 15 years or older, new case or relapse, who were diagnosed with active tuberculosis, defined according to WHO criteria [14] and had started the TB treatment according to national guidelines [15] between January the 1rst 2009 and December 31rst 2010. Patients who refused to take part in the survey were excluded. The sample size was calculated taking into account prevalence of TB treatment defaulter with an alpha error of 5% and power of 80%.

### Study conduct and data collection

All TB treatment defaulters in 2009 and 2010 identified in the TCUs were selected for the study. Data were collected by nurses using anonymous questionnaire. The nurses were staff members of the TCUs and were responsible of actively searching for cases and controls to collect the data. A French version of questionnaire was developed, validated and pre-tested by the Department of Epidemiology in Fez University. It was also translated into Moroccan Arabic dialect. Adjustments were made after assembling a focus group of all nurses participating in the study. Participants were interviewed in the TCUs. The questionnaires was administered verbally, it contained multiple-choice and open-ended questions about factors that might be associated with default such as:

Socio-demographic factors: sex and age, educational level, housing area and conditions, monthly earnings; Knowledge on TB: cause and transmission, consequence of stopping treatment and its duration, Attitudes related to TB; subjective reasons for defaulting; global evaluation of the health system with questions on satisfaction with different aspects of the medical care.

### Data analysis

Descriptive statistics were used for patients’ demographic information, knowledge, and attitudes and toward TB and it consisted of frequency counts and percentages. Quantitative data was summarized as mean +/- SD or 95% Confidence Interval. Qualitative data was summarized as percentages. Chi-square test was used to test the difference in proportions of socio- demographic variables, knowledge and attitude according to treatment adherence. The level of statistical significance was defined as p

### Ethical consideration

The survey underwent ethical review. The purpose was explained in details to each patient agreeing to participate in the study, and oral informed consent was obtained prior to interviews. Anonymity of questionnaires was strictly respected.

## Results

### Respondents profile

An interview was obtained from 94.8% of the 306 patients targeted. Main reasons for not achieving an interview included refusal due to disinterest or unwillingness. Among the 16 patients who did not agree to participle, 12 were defaulters and 10 were men. Concerning the 290 patients included for analysis, 68.8% of them were men and the mean age was 31.7 ± 12.0 years; 56.4% were aged ≤30 years. Regarding clinical form of TB, approximately 7 cases in 10 were pulmonary TB and cases 9 cases in 10 were new TB cases. Monthly income was under 2000 MAD (180 €) for 82% of respondents and 61.0% were unemployed or had an occasionally activity. Over sixty four percent were illiterate or had a basic educational level.

Table 1 shows a comparison between adherent patients and non-adherent patients. The groups showed no significant differences regarding monthly income. Of the socio-demographic characteristics measured, the difference concerning professional activity and educational level were significantly associated with non adherence. Proportion of unemployed was significantly higher among non-adherent patients: 74.1% versus 55.6% (p =0.003). Non-adherent patients were less educated (<6 years) than adherent patients (p<0.05). Walking distance to nearest treatment centre was more than 15 minutes for 61.2% of respondents; 75.3% of non-adherent patients versus 55.4% of adherent patients (p=0.001).

**Table 1:** Comparison between adherent and non-adherent patients by socio-demographic variables

Variables	%	Adherent patients	Non-adherent	p
**Monthly income** 1 **(N=270)**				**0.06**
<180 €	81.7	79.0	88.2	
≥180 €	18.3	21.0	11.8	
**Socio-professional category (N=289)**				**0.003**
Unemployed or Occasional activity	61.0	55.6	74.1	
Permanent activity	39.0	44.4	25.9	
**Educational level (N=263)**				**<0.05**
<6 years	64.3	60.4	72.8	
≥ 6 years	35.7	39.6	27.2	

1 family income

### Respondents′ general knowledge about TB and its treatment

Cough was the most commonly stated symptom in respondents (48.3%). Other symptoms cited were coughing up blood (36.8%) and expectoration (36.1%). There were no significantly difference between adherent patients and non-adherent patients. Table 2 illustrates the knowledge of the TB patients about their disease according to adherence status. Out of the total number of interviewees, 53.8% knew that TB is a contagious disease, less than half (45.1%) knew that TB is transmitted through air and 15.7% reported that TB is caused by tobacco; 27.3% among non-adherent patients versus 8,7% among adherent patients (p<0.001). The microbial cause of infection was only known by 17.2% of the respondents; 20.5% among non-adherent patients versus 9.4%% among non-adherent patients (p=0.02).

**Table 2:** Knowledge of the TB patients about their disease and the cause according to adherence status

Knowledge items: cause	Total Frequency %	Adherent patients %	No adherent %	p
TB is caused by a microbe (N=290)	17.2	20.5	9.4	0.02
TB is a contagious disease (N=285)	53.8	54.4	52.4	NS
TB is caused by tobacco (N=261)	15.7	27,3	8.7	<0.001
TB is transmitted through air (N=288)	45.1	47.1	40.5	NS
TB can easily be cured these days if you take your treatment (N=289)	95.8	99.0	88.2	<0.01
Duration treatment (N=290)				
Six than nine months	83.8	85.9	82.9	NS
**Consequences of not completing treatment**				
Can't be cured (N=285)	61.4	63,5	56,5	0.06
Relapse (N=285)	16.1	22.4	13.5	0.04
Dying (N=285)	53.5	48.2	58.2	NS
No idea (N=290)	3.8	1.5	9.4	0.001
**Patient–provider communication**				
Well explication about disease (N=288)	79.2	84.2	67.1	0.001
Well explication about treatment duration and stopping consequence (N=268)	83,5	89,0	69,7	<0.001
Walk distance from health facility >15 min (N=289)	61.2	55.4	75.3	0.01

The duration of TB treatment was well known by both adherent and non-adherents patient (83.8%), and regarding the fact that the disease is curable if treatment is taken was known by 95.8% of the respondents; this proportion was significantly higher among adherent patients: 99.0% versus 88.2% among non-adherent patients (p<0.01). Concerning the consequences of not completing treatment, 3.8% of respondents has no idea; 9.4% among non-adherent patients versus 1.5% among adherent patients (p<0.01), and 16.1% believe that stopping treatment is a cause of relapse; 22.4% among adherent patients versus 13.5% among non-adherent patients (p<0.05).

Adherent patients were more informed by health care workers about their disease; 84.2% versus 67.1% among non-adherent patients (p=0.001). Regarding information about treatment, eighty tree percent of patients had been informed by health care workers (HCW) about consequences of not completing treatment and its duration, the proportion was significantly higher adherent patients: 89.0% versus 69.7% among non-adherent patients (p<0.001).

### Reasons for defaulting

The first reason why TB patients stop taking their treatment before they are cured is “feeling better” which was reported by 72.9% of no adherent patients, followed by “long duration of treatment” (34.1%). “Side effect” and “difficulty to access to health facility” were reported respectively by 9.4% and 4.7% of non-adherent patients.

### Attitude and satisfaction with services

Table 3 shows attitude and satisfaction with service according to adherence status. Concerning attitude, 82.4% of respondents said that TB patient can stop its treatment if he feels better; 93.3% among non-adherent patients versus 65.5% among adherent patients (p=0.02). More than half of patients (57.9%) said TB patient can stop DOTS it if he can't support medication, and 76.5% said there are many treatment to take. 64.2% believes that if you have TB people do not respect you. There were no significant difference between adherents and non-adherent's patient. Ninety one percent of respondents were satisfied with health facilities, the proportion was significantly higher among adherent patients: 94.1 versus 84.5 among non-adherent patients (p<0.01).

**Table 3:** Attitude toward TB and satisfaction with services

Items	Frequency %	Adherent patients %	No adherent %	p
Attitude toward TB				
Respondent believes that if you have TB people do not respect you	64,2	64.4	63.9	NS
Patient can stop its treatment if he feels better (N=74)	82.4	65.5	93.3	0.02
Patient can stop its treatment if he can't support medication (N=38)	57.9	58.3	57.1	NS
Many treatment to take (N=298)	76.5	75.6	78,6	NS
Satisfaction with services (N=289)				
Satisfied patients	91.3	94.1	84.5	<0.01

## Discussion

This study is the first to explore knowledge, attitude and treatment default in Fez region. Despite the patient provider interaction, TB knowledge can be considered fairly poor among this community. Less than half of the patients (45.1%) knew that TB is transmitted through air. More grave was the fact that only 17.2% known that it is caused by a microbe. Respondents were poorly informed about consequence of stopping treatment. Conversely, there were well informed about treatment duration.

Our finding on the cause of TB is higher than a recent study data of knowledge of TB in urban Morocco which showed that only 9% of non-patients attribute TB to an infectious cause [16]. Other studies in developing countries have demonstrated misconceptions regarding TB among TB patients. In Pakistan, patients who had already visited healthcare providers and initiated their treatment showed lack of knowledge on their disease [17], and in Turkey, patients expressed unhappiness and stress (23.7%) to be the major cause of their illness [18]. The basic patient's knowledge about transmission and consequence of not completing TB treatment are consistent with the studies elsewhere [19]. Our results suggest a lack of quality communication between the health workers and the patients.

Majority of the respondents, both adherent patients and non-adherent patients, indicated that they would feel embarrassed and ashamed if they learned they had TB. Similar feelings have long been associated with TB [20]. Community stigma stems from a perceived risk of infection and perceived link between TB and poverty, low caste, disreputable behavior and divine punishment. It can be further explained by the values deep rooted in the cultural fabric of Morocco, where a TB patient has long been condemned, disgraced and marginalized by the society.

The main reason for not completing the treatment was the impression of being cured. Several studies have reported feeling cured as the main reason for defaulting. In Zambia, the major factor leading to non-adherence included patients beginning to feel better (45.1%) [21], and in Indonesia, feeling better was the most frequently mentioned reason (47%) [22]. In our study, 73.9% of defaulters evoked this reason to interrupt their treatment, suggesting the lack of explanations on the treatment. Considering that patients start feeling well after only a few weeks of treatment, it is of the utmost importance to explain to patients the importance of completing their treatment although they don't feel the need for it and might experience side-effects. To minimize defaulting, patients should be given comprehensive counseling at start of treatment of the disease, with sufficient explanation on the disease, its treatment and also possible side-effects and the availability of means to manage them.

Poor awareness especially regarding symptoms and treatment results and consequence were reported to have been associated with treatment non-completion, in accordance with other settings results [23]. Perception of stopping treatment (to believe that TB patient can stop its treatment if he feels better) was a determinant for treatment default in consistence with South Africa data [24]

Other risk factors such as, poor patient-health provider communication, insatisfaction with service and distance from health facility (> 15 min walking) were apparent determinants of default and barriers in utilization of services. Lack of support from health staff, dissatisfaction, and decentralization of treatment associated with treatment success has been demonstrated in studies in Kenya [25].

This finding is interesting and justifies the need for improved communication and mutual understanding between care providers and patients. Implementing of interventions aimed at improving communication and mutual understanding between care providers and patients into this matter by the national tuberculosis program would improve TB treatment outcomes nationally.

TB patients′ experiences are helpful at organizational/institutional and community level in developing training programs and new interventions that should contribute to stigma reduction rather than unintentionally enhance stigmatization.

These findings allow recommendations to be formulated concerning management of TB patients regarding the risk of drug default, however focus should not only be on patients but also on health services, ensuring that they are appropriately adjusted to patients’ needs.

There is a definite need to make accessible time and place of the TB clinics more convenient and flexible for patients.

In addition to comprehensive explanations on the disease itself, patients should be informed about the side-effects of the drugs, the duration of DOTS and the consequences of interrupting treatment. A study on South Africa demonstrated the effectiveness of training the nursing staff in communication skills, resulting in mutual satisfaction in patients and healthcare providers [26]. Such measures should be introduced in the actual training of TB healthcare workers in Morocco.

Questioning each patient at start of treatment on addictive behaviors should be part of routine evaluation, and specific substance abuse programs within TB services to encourage tobacco and alcohol cessation in TB patients should be developed. A recent study in Morocco shows that tobacco is a risk factor of TB treatment default [11].

Patients living far from the TCU should be considered at risk of defaulting and though TB care is already free of charge, it might be useful to introduce public transportation passes to get free transportation to the closest TCU as an integral part of TB care.

However, some limitations need to be pointed out. First, interviews were carried out by the nurses working in the health centers. Consequently, all questions that tried to evaluate relations and interaction with the healthcare workers might have led to interview bias, nurses possibly leading questions and respondents wishing to evade or lie to some questions. Untruthful answers were likely to have been limited by the thorough explanations given by the nurses before interviews and complete anonymity of all questionnaires. Another limitation that needs to be discussed is the retrospective nature of the collected data. Participants were treated more than two years ago and might have been subject to recall bias. However, some information lost due to recall was completed to a certain extent by the review of the medical records.

## Conclusion

Default to TB treatment is a major barrier to optimal care of TB patients, and an important challenge in the national TB control program. Completion of treatment is the necessary condition for the patient's cure and the prevention of multi-resistant TB strains. This study explored factors for TB treatment default in Fez, Morocco and identified objective and subjective reasons for defaulting. Reducing the number of defaulters is possible through realistic measures such as reinforcing quality communication between patients and providers, implementing programs to treat addictive behaviors and increasing accessibility to the TCUs. Such measures are likely to increase the therapeutic success rate, impacting on global disease burden attributable to TB in Morocco.
